# Anatomic Conformation of Renal Sympathetic Nerve Fibers in Living Human Tissues

**DOI:** 10.1038/s41598-019-41159-4

**Published:** 2019-03-18

**Authors:** Won-Seok Choe, Won Hoon Song, Chang Wook Jeong, Eue-Keun Choi, Seil Oh

**Affiliations:** 1 Department of Internal Medicine, Seoul National University Hospital, Seoul, Republic of Korea; 2 Department of Urology, Pusan National University Yangsan Hospital, Yangsan, Republic of Korea; 3 Department of Urology, Seoul National University Hospital, Seoul, Republic of Korea

## Abstract

Renal denervation using radiofrequency catheter ablation is known to eliminate the renal sympathetic nerve and to lower blood pressure in patients with resistant hypertension. We sought to investigate the detailed anatomic conformation of the peri-renal arterial sympathetic nerve fibers with living human specimens. Peri-renal arterial tissue was harvested from patients undergoing elective radical or simple nephrectomy. Digital images of each section from the distal arterial bifurcation to the proximal margin were obtained and analyzed after immunohistochemical staining with anti-tyrosine hydroxylase antibodies. A total of 3,075 nerve fibers were identified from 84 sections of peri-renal arterial tissue from 28 patients (mean age 62.5 ± 10.2 years, male 68%). Overall, 16% of nerve fibers were located at distances greater than 3 mm from the endoluminal surface of the renal artery. The median distance from the arterial lumen to the nerve fibers of the proximal, middle, and distal renal arterial segments was 1.51 mm, 1.48 mm, and 1.52 mm, respectively. The median diameter of the nerve fibers was 65 μm, and there was no significant difference between the segments. A substantial proportion of the sympathetic nerve fibers were located deeper in the peri-arterial soft tissue than in the lesion depth created by the conventional catheter-based renal sympathetic denervation system.

## Introduction

Hypertension affects approximately 40% of the adult population worldwide^1^, causing an enormous disease burden with complications of cardiovascular morbidity and mortality^2^. Although pharmacologic anti-hypertensive therapy is generally effective, a significant proportion of patients fail to achieve full control of blood pressure despite being prescribed a combination of medications^3^. These challenges have raised interest in developing an alternative, device-based approach to hypertension management.

The sympathetic nervous system is a key component in the development of cardiovascular disease, and especially in hypertension and heart failure^4^. The kidneys are richly innervated with the sympathetic nerve fibers, which play a pivotal role in hypertension by stimulating renin secretion and tubular sodium reabsorption and by reducing urinary sodium excretion^5^. While surgical sympathectomy fell out of favor due to significant perioperative morbidity and adverse effects^6,7^, introduction of a minimally invasive catheter-based renal sympathetic denervation (RDN) facilitated clinical application of sympathetic denervation and promoted further research. Early clinical studies suggested promising results, and catheter-based RDN provided a significant reduction in blood pressure with an acceptable safety profile, even in patients with resistant hypertension^8–11^. However, a large, blinded, randomized, and sham-procedure controlled trial (SYMPLICITY HTN-3) did not show a significant difference in the reduction in blood pressure between the renal denervation and sham-procedure groups^12^. This report was striking given the sound pathophysiological base of RDN, and had a detrimental effect on the prospect of RDN.

The results of SYMPLICITY HTN-3 became the focus of extensive debate and provoked efforts to identify and supplement the factors contributing to the failure^13–17^. As a result, there was a renewed interest in the renal nerve anatomy and physiology, and a substantial number of studies on the anatomy of the renal artery and peri-renal sympathetic nerve have since been published. Nevertheless, the histological data on human anatomy remain limited and are primarily based on post-mortem samples^18–21^. Previous studies have shown that gross vascular structure and peripheral nerve histology have substantial changes during the post-mortem period^22,23^, and results from post-mortem samples may not accurately reflect *in vivo* peri-arterial renal nerve distribution. In the current study, we aimed to conduct a morphometric analysis of the sympathetic innervation of human renal arteries with specimens from living patients who underwent elective nephrectomy.

## Methods

This study is based on clinical information, computed tomography data, and histological analysis of renal arteries and surrounding peri-renal soft tissue obtained from 100 consecutive patients who underwent elective radical or simple nephrectomy between April 2014 and July 2016 at Seoul National University Hospital. The study was conducted in accordance with the ethics principles in the Declaration of Helsinki and applicable amendments, and the study protocol was approved by the Institutional Review Board of Seoul National University Hospital Biomedical Research Institute (No. 1503-087-657). All participating patients provided written informed consent.

### Tissue preparation

**Figure 1:**
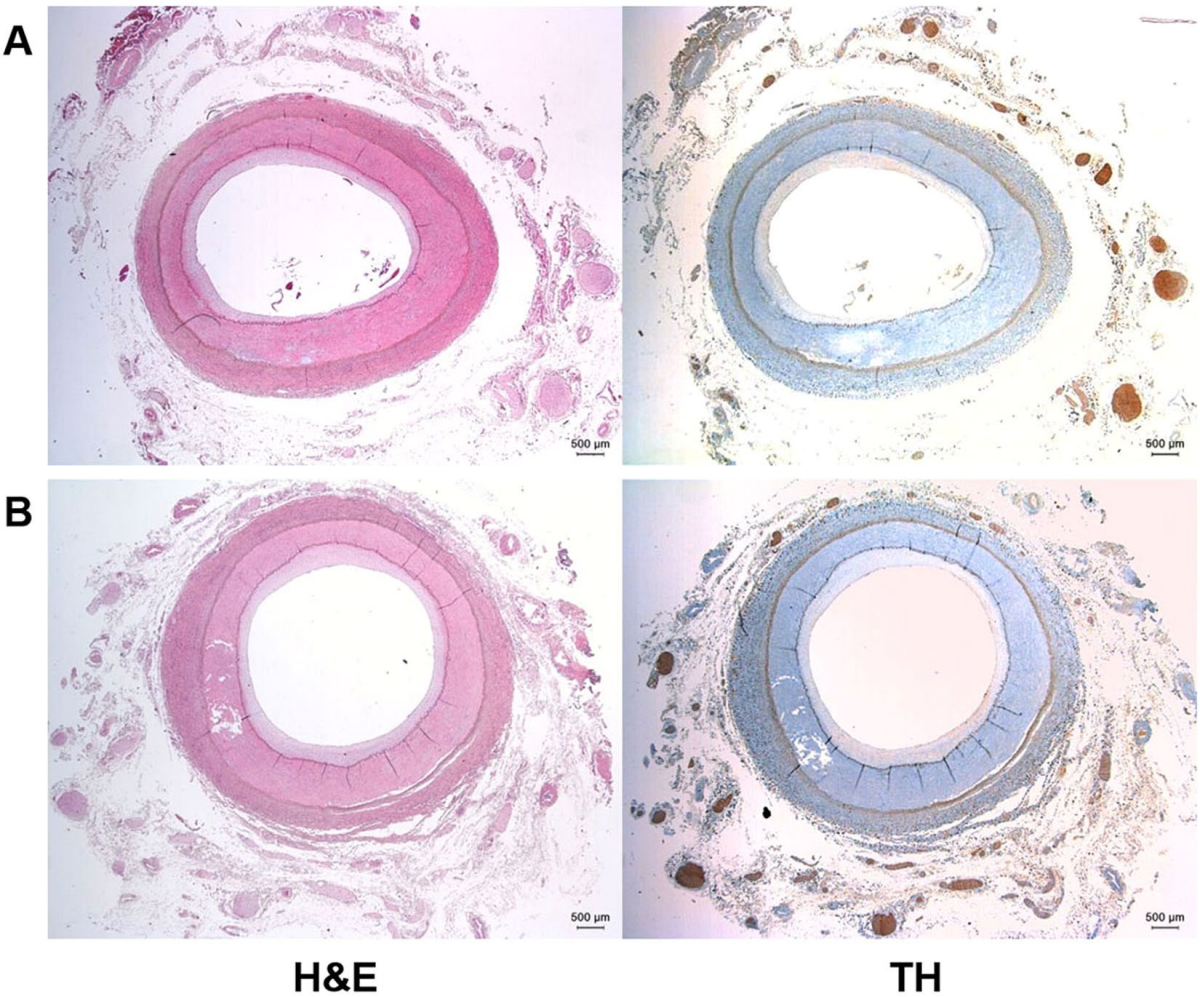
Representative images of renal artery segments. Cross sections of the (**A**) proximal and (**B**) distal segments of renal artery and peri-renal soft tissue. Images on the left are hematoxylin and eosin (H&E) stained, and images on the right are stained for tyrosine hydroxylase (TH).

Renal arteries were harvested en bloc for as long as possible during nephrectomy. The distal part of renal artery ligation and the surrounding hilar structures including the renal vein were kept intact during the surgery and tissue preparation. The distal portion of the vessels were transacted at entry level to the renal parenchyma. Despite our extensive efforts to not deform the tissue, a proportion of samples were harvested with damage to the gross structure and were therefore excluded from the histological analysis. Subsequently, 28 intact specimens from the vascular clamping site of the proximal renal artery to the distal part of early arterial bifurcation were isolated. Peri-renal tissue was fixed in a 4% buffered solution of para-formaldehyde for 24 hours, while the proximal and distal ends of renal artery were sealed and filled with fixative solution at a pressure of 80 mmHg to maintain the luminal structure. All preparations were made to include the surrounding tissue with renal arteries, and the fixed specimens subsequently underwent delipidation, dehydration, and paraffin embedding for measurement. Tissue blocks containing renal artery and peri-renal arterial tissue were sectioned perpendicularly to the longitudinal axis from the distal early arterial bifurcation to the proximal margin. Among these tissue sections, the proximal arterial ends with anatomical distortion by vascular clamp and the distal ends with elliptical cross-sectional geometry due to adjacent bifurcation were excluded from the analysis. Depending on the proximity to the aorta, three representative sections with equal longitudinal intervals were selected for comparative anatomical analysis and were assigned to proximal, middle, and distal segments, respectively. In addition to the standard hematoxylin and eosin staining, immunohistochemistry using tyrosine hydroxylase was performed for each section to localize the peri-arterial sympathetic nerve fibers (Fig. 1).

### Histological analysis of renal arteries and peri-renal nerve fibers

**Figure 2:**
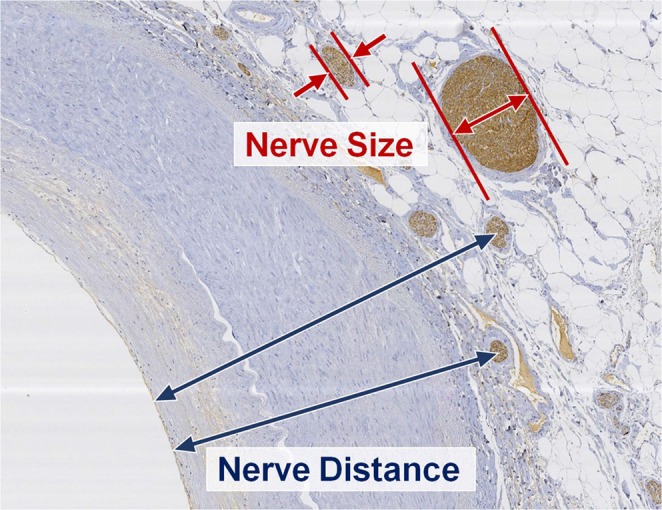
Measurements of peri-renal arterial sympathetic nerve distribution. Nerve distance was measured from the closest endoluminal surface of the renal artery to the center of the nerve fiber. The smallest diameter of nerve fiber was measured as a representative value of nerve size to prevent overestimation in case of oblique sectioning during tissue preparation.

Digital images from histological sections were acquired, and anatomical measurements were made using Leica Application Suite image analysis software (Leica Biosystems, Wetzlar, Germany). All nerve fibers were identified and morphometric analysis was performed to quantify the following parameters: total number of nerves per section, nerve distance (distance of each nerve to the closest endoluminal surface of the renal artery), and radial diameter of each nerve fiber. The representative images showing methods of morphometric measurement of peri-arterial nerve fibers are shown in Fig. 2. While our primary analysis was performed on the distribution of nerve fibers in the entire tissue samples, individual patient level data were also visualized using Tableau (Tableau Desktop Professional Edition 10.1.1).

The cross-sectional structures of the renal arteries were also analyzed in each section using the following parameters: mean luminal diameter (the average of the maximum and minimum luminal diameter of each section), luminal area, mean external elastic membrane (EEM) diameter, EEM area, and mean wall thickness (the average of the wall thickness from the luminal surface to the EEM measured in 4 quadrants of the renal artery).

### Imaging analysis of renal arteries

We analyzed the CT image of the patients to reveal the gross anatomy of the renal arteries and supplement histological data, which is confined to the main trunk of the renal arteries. Imaging analysis included all participating patients (n = 100) with available CT data, and the length, diameter, and branching pattern of the bilateral renal arteries were assessed. In the imaging analysis, the accessory renal artery was defined as the sub-artery branched directly from the aorta, and the accessory branched artery was defined as the sub-artery branched early (within 2.0 cm of aorta) from the main renal artery.

### Statistical analysis

Baseline characteristics are expressed as mean ± standard deviation for continuous variables and as number and percent for categorical variables. Measured values of each nerve fiber were used individually in the tables and figures for the total distribution. Matched comparison of nerve counts corresponding to the proximal, middle, and distal arterial segments was performed using a repeated measures ANOVA, and comparisons of nerve distance and size were performed using a linear mixed model, using arterial segment as fixed effect and study subject as random effect. A p-value of <0.05 was regarded as significant. Statistical analyses were performed using SPSS version 23 (IBM Corporation, Chicago, IL, USA).

## Results

A histological analysis of the renal arterial anatomy and sympathetic nerve distribution was performed in a total of 84 arterial segments from 28 patients. The characteristics of the patients included in the histological analysis are summarized in Table 1. The mean age of the patients was 62.5 years, and 32.1% were female. The majority of the patients underwent nephrectomy due to underlying malignancies.

**Table 1:** Demographic and clinical characteristics of study patient.

	Total patient population (n = 28)
Age, yrs	62.5 ± 10.2
Female	9 (32.1%)
BMI	23.3 ± 2.9
Indication of surgery (malignancy/non-malignancy)	25/3
Extracted kidney (right/left/unknown*)	12/15/1
Hypertension, %	16 (57.1%)
Diabetes mellitus, %	8 (28.6%)
Dyslipidemia, %	5 (17.9%)
Coronary artery disease, %	4 (14.3%)
Heart failure, %	4 (14.3%)
Estimated GFR (eGFR)	
Mean eGFR, ml/min/1.73 m^2^	56.0 ± 25.2
eGFR less than 60 ml/min/1.73 m^2^	13 (46.4%)
Concurrent medication	
ACE inhibitor or ARB, %	12 (42.9%)
Beta blocker, %	6 (21.4%)
Diuretics, %	5 (17.9%)

Plus-minus values are mean ± SD.

*One patient underwent bilateral nephrectomy, and the laterality of the harvested peri-renal arterial tissue could not be confirmed retrospectively.

Abbreviations: ACE, angiotensin converting enzyme; ARB, angiotensin receptor blocker; BMI, body mass index; GFR, glomerular filtration rate.

### Renal arterial size

The renal arterial anatomy of each segment is summarized in Table 2. Mean lumen diameter between the proximal, middle, and distal segments was 2.2 ± 1.1 mm, 2.3 ± 1.2 mm, and 2.4 ± 1.1 mm, respectively. No significant differences were found in the luminal diameter (p = 0.874), EEM diameter (p = 0.665) and wall thickness (p = 0.975) between the segments with different proximities.

**Table 2:** Measurements of renal artery in study patients.

	Total	Segments			
	(n = 84)	Proximal (n = 28)	Middle (n = 28)	Distal (n = 28)	p value
Mean lumen diameter,* mm	2.31 ± 1.11	2.24 ± 1.12	2.32 ± 1.15	2.38 ± 1.10	0.874
Lumen area, mm^2^	4.72 ± 4.57	4.65 ± 4.80	4.69 ± 4.64	4.82 ± 4.41	0.989
Mean EEM diameter, mm	3.61 ± 1.24	3.47 ± 1.22	3.60 ± 1.28	3.75 ± 1.26	0.665
EEM area, mm^2^	11.16 ± 7.53	10.84 ± 7.82	11.09 ± 7.90	11.55 ± 7.08	0.931
Mean wall thickness^†^, mm	0.62 ± 0.18	0.62 ± 0.18	0.63 ± 0.18	0.63 ± 0.17	0.975

Plus-minus values are mean ± SD.

*The average of the maximum and minimum luminal diameter.

^†^The average of the wall thickness from the luminal surface to the EEM measured in 4 quadrants.

Abbreviations: EEM, external elastic membrane.

### Total nerve count

A total of 3,075 nerve fibers were located around the renal arteries. Total nerve count gradually increased from proximal to distal segment, and the average total nerve count of the proximal, middle, and distal segments was 32.8 ± 14.9, 35.7 ± 12.1, and 41.3 ± 17.9, respectively (p = 0.048). The average total nerve count per section was similar in the right renal artery (35.8 ± 13.2) and left renal artery (36.4 ± 17.2). However, statistical comparison between the right and left renal artery is limited as each sample was obtained from different individual patients.

### Nerve distribution by distance

**Figure 3:**
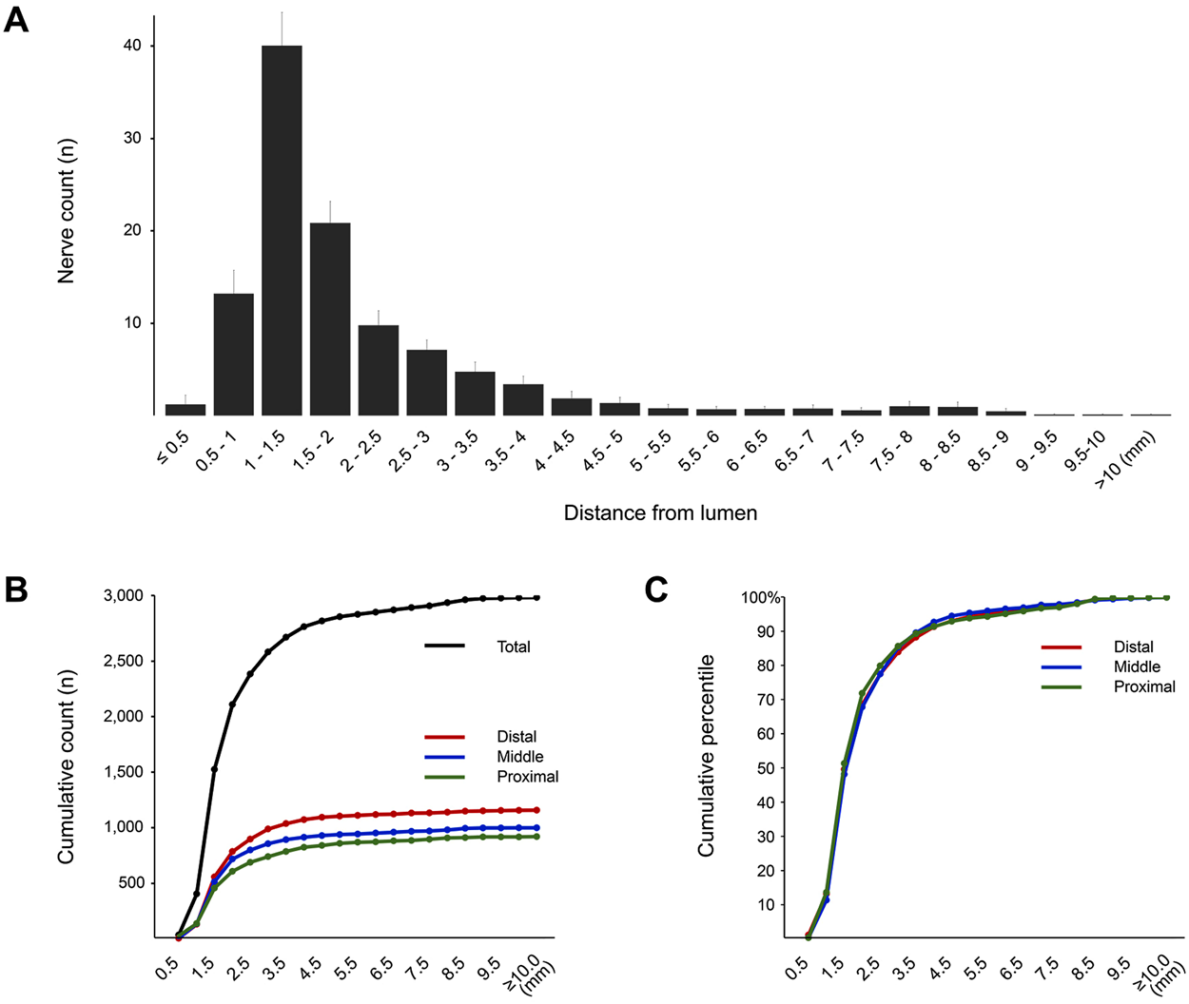
Distribution of nerve fibers and distance from the endoluminal surface. (**A**) The average of nerve fiber counts was stratified according to the distance from the endoluminal surface. (**B**) The cumulative absolute and (**C**) relative distribution of nerve fibers was divided into the proximal, middle, and distal segments. Overall, 68.6%, 84.0%, and 91.4% of the total nerve fibers were located within 2, 3, and 4 mm from the lumen of the renal arteries. The spatial distribution of nerve fibers in terms of median distance was not significantly different between the proximal and distal segments of renal artery.

**Figure 4:**
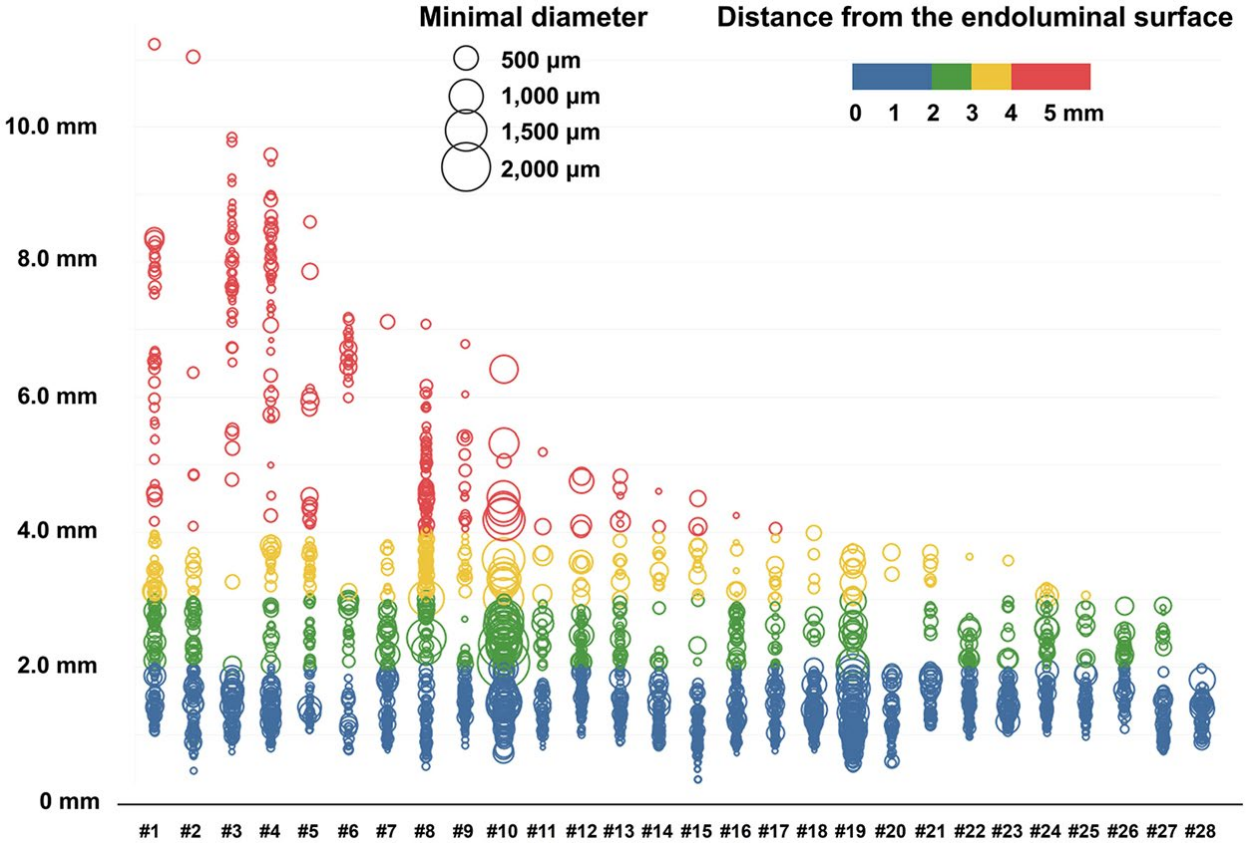
Distribution of the peri-renal arterial sympathetic nerve fibers in individual patients. Each circle represents one nerve, the size of circle indicates the diameter of nerve fiber, and the color of circle shows the distance from the luminal surface of renal artery to nerve fiber.

The overall distribution of peri-renal sympathetic nerves stratified by the distance from the arterial endoluminal surface of each segment is described in Table 3, and Fig. 3. Overall, 68.6%, 84.0%, and 91.4% of the total nerve fibers were located within 2, 3, and 4 mm from the lumen of the renal arteries. The median distance of entire individual nerve fibers was 1.50 mm, and the spatial distribution of nerve fibers in terms of median distance was not significantly different between the proximal and distal segments of the renal artery (p = 0.368). Figure 4 comprehensively presents the distribution of total nerve fibers in individual patients, and Supplementary Fig. 1 shows the same data with subdivision into each segment.

**Table 3:** Distribution of peri-renal sympathetic nerves stratified by the distance from the arterial endoluminal surface in proximal, middle, and distal segments of renal artery.

	Total	Segments			
	(n = 3,075)	Proximal (n = 919)	Middle (n = 999)	Distal (n = 1,157)	p value
Distance from endoluminal surface, mm					
<1.0	404	137	136	131	
≥1.0 < 2.0	1,706	470	582	654	
≥2.0 < 3.0	473	132	138	203	
≥3.0 < 4.0	228	85	58	85	
≥4.0 < 5.0	90	35	24	31	
≥5.0 < 6.0	41	14	13	14	
≥6.0 < 7.0	41	12	16	13	
≥7.0 < 8.0	44	23	13	8	
≥8.0 < 9.0	39	9	18	12	
≥9.0 < 10.0	6	0	1	5	
≥10.0	3	2	0	1	
Median distance, mm	1.50	1.51	1.48	1.52	0.712

Values are n or median. Comparisons of nerve distribution between different segments were performed using a linear mixed model.

There was no significant difference in nerve fiber distribution between subjects with and without a history of hypertension. (Supplementary Fig. 2) We also analyzed the nerve distance of the bilateral renal arteries individually (Supplementary Fig. 3). The overall median nerve distance was 1.37 mm in the right side (1.36 mm, 1.37 mm and 1.36 mm for proximal, middle and distal segments, respectively), and 1.58 mm in the left side (1.64 mm, 1.54 mm and 1.59 mm for proximal, middle and distal segments, respectively). However, direct comparison of the spatial distribution of bilateral peri-renal arterial nerve was not feasible, because each sample was obtained from different patients and the sample size was relatively small.

### Nerve distribution by size

**Figure 5:**
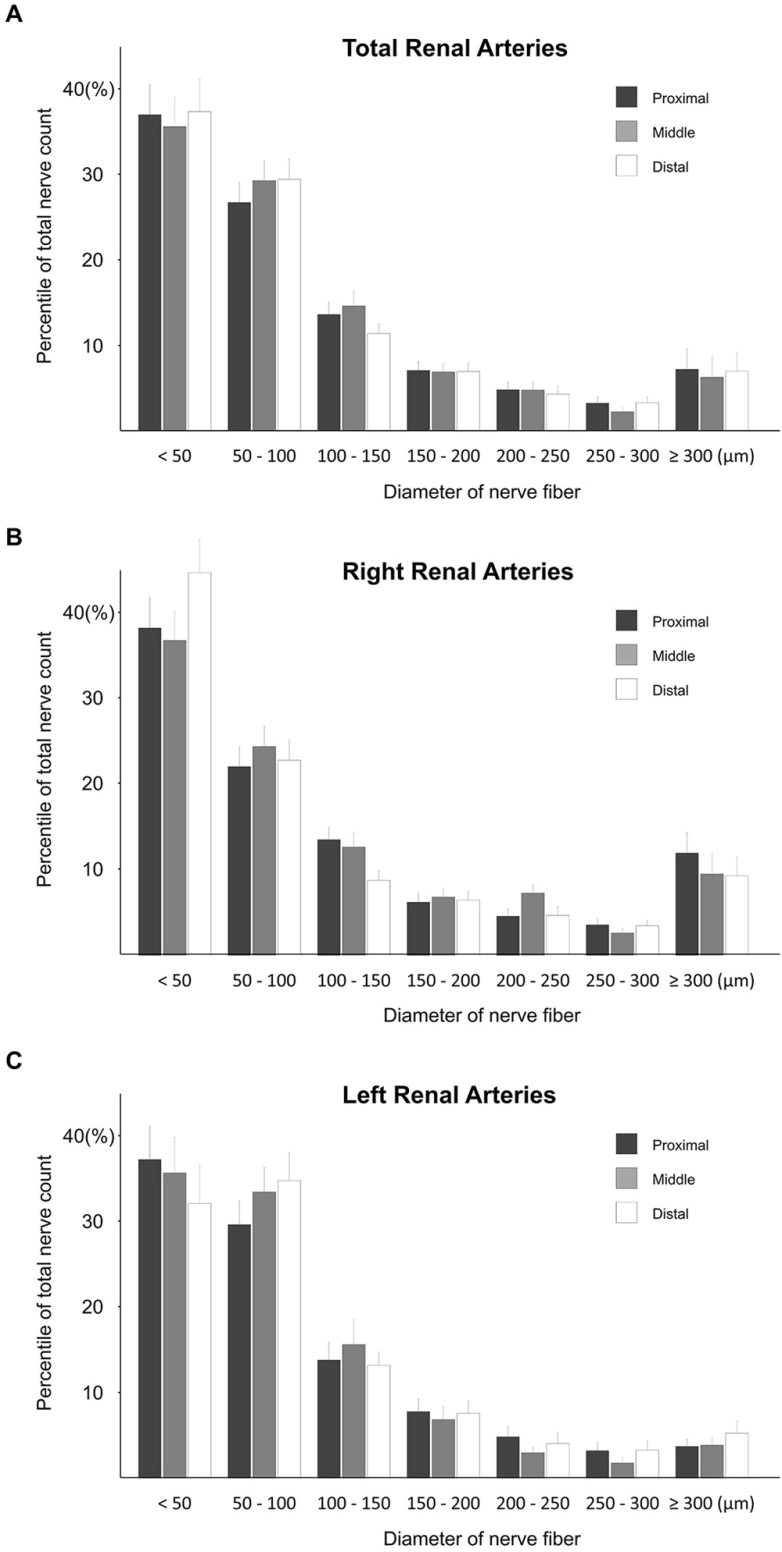
The percentage of total nerve counts surrounding renal arteries according to the proximity within the arterial length. (**A**) Overall distribution of nerve fibers was divided into the proximal, middle, and distal segments. Data were dissected into arterial segments for the right (**B**) and left (**C**) renal arteries.

Table 4 and Fig. 5 shows the size distribution of peri-arterial sympathetic nerve fibers. In the current analysis, the smallest diameter of each nerve fiber was measured as a representative value of nerve size to prevent overestimation in case of oblique sectioning during tissue preparation. Of the total nerve fibers, the median nerve diameter was 65 μm and 90% of the nerves were less than 250 μm in diameter. With a pooled analysis integrating data from all study subjects, nerve fiber size showed a tendency to decrease gradually toward the distal segment and the median diameter of individual nerve fibers between the proximal, middle, and distal segments was 70 μm, 66 μm, 61 μm, respectively. However, when comparing the paired observations from each subject, no significant differences in nerve size between renal arterial segments with different proximities to the aorta were observed (p = 0.704). This tendency was confirmed in renal arteries on both sides (Supplementary Tables 1 and 2).

**Table 4:** Distribution of peri-renal sympathetic nerves stratified by the size of nerve fibers in proximal, middle, and distal segments of renal artery.

	Total	Segments			
	(n = 3,075)	Proximal (n = 919)	Middle (n = 999)	Distal (n = 1,157)	p value
Diameter of nerve fiber, μm					
<50	1177	333	372	472	
50–100	860	250	278	332	
100–150	386	123	141	122	
150–200	210	65	71	74	
200–250	145	48	48	49	
250–300	84	28	23	33	
300–350	51	18	17	16	
350–400	45	18	8	19	
400–450	26	6	10	10	
450–500	16	4	7	5	
≥500	75	26	24	25	
Median diameter, μm	65.0	70.0	66.0	61.0	0.449

Values are n, % or median. Comparisons of nerve distribution between different segments were performed using a linear mixed model.

### Imaging analysis

Supplementary Table 3 shows the characteristics and distribution of renal artery by CT abdomen review in 100 of patients who underwent nephrectomy. Overall, 83% of patients had bilateral single renal arteries, while the remaining patients had at least one accessory renal artery or accessory branched artery. Supplementary Fig. 4 demonstrates the outer diameter of each segment of the main renal artery from the aorta to bifurcation via a CT abdomen review in 100 of patients who underwent nephrectomy. The outer diameter of the renal artery was decreased from the bifurcation point of the main renal artery to 3, 6, and 9 mm to the proximal segment. In addition, the outer diameter of the renal artery was decreased from the proximal main renal artery to the distal segment.

## Discussion

To the best of our knowledge, this is the first study analyzing the peri-renal sympathetic nerves from living human renal arteries. The main findings of our study are as follows: (1) peri-renal sympathetic nerves showed a skewed distribution with a median distance of 1.5 mm from the endoluminal surface of renal arteries; (2) approximately 16% of nerve fibers were located at distance greater than 3 mm; and (3) there was no significant difference in the spatial distribution of nerve fibers in terms of distance from renal arteries and size according to the proximity of the renal arterial segments, while the number of nerve fibers tended to increase toward the distal segments.

RDN for the treatment of hypertension has undergone considerable changes over the last decade. While the results of the SYMPLICITY HTN-3 trial waned enthusiasm raised from the striking efficacy of RDN in earlier, uncontrolled trials, there has also been many debates about these unexpected results. Technical issues including operator inexperience^13^, suboptimal methods and devices used in RDN procedures^14,24,25^, and the innate limitations coming from anatomical substrate^20,26,27^ have been major concerns in this field. Among these considerations, accurate understanding of the anatomical patterns of renal artery innervations could lead to refinements in RDN procedures and possibly improve clinical outcomes.

### Anatomical considerations in renal sympathetic denervation

The distance from the energy source to the target structure is one of the main determinants of the therapeutic efficacy of radiofrequency ablation, and nerve fibers located in distant tissue cannot be effectively ablated with current RDN procedures. There are several histologic studies reporting peri-renal innervation of sympathetic nerves with post-mortem human samples. Atherton *et al*. reported that more than 90% of sympathetic nerve fibers were localized within 0.5–2 mm depth from the lumen of the renal artery, but they limited their analysis to the tissues only within 2.5 mm distance from the renal arterial lumen^18^. Sakakura *et al*. analyzed a broader range of peri-renal arterial tissue, and reported that 40.7%, 58.5%, and 71.6% of the total nerve fibers were located within 2, 3, and 4 mm distances^19^. Van Amsterdam *et al*. also investigated peri-renal arterial tissue upto 7 mm depth and reported 58.8% of total nerve cross-sectional area to be within 2 mm from the arterial lumen^28^. Although peri-arterial sympathetic nerves are distributed in proximity to the renal artery, a substantial proportion of nerve fibers are located outside the range of 2 to 4 mm. In line with these studies, we found that only 1 (3.6%), 3 (10.7%) and 11 (39.3%) of 28 subjects had all observed renal sympathetic nerve fibers within 2 mm, 3 mm and 4 mm from the endoluminal surface of the renal arteries, respectively (Fig. 4). It is interesting that the renal arterial size and per-arterial nerve distribution in hypertensive subjects were not significantly different from those in non-hypertensive subjects. Sympathetic hyperactivity is a well-known risk factor for the development of hypertension^29^, but data on the direct relevance of the peri-arterial renal sympathetic nerve distribution to hypertension are limited^19^. The clinical significance of the current observation should be determined with further studies.

Previous studies that examined the renal innervation in post-mortem human samples suggested that the renal nerves form a plexus with a wide base directed toward the aorta and apex converging toward the kidney, so that the average distance from the lumen to the nerve was longest in the proximal segment and shortest in the distal segment^19,28^. This hypothesis is inconsistent with our analysis, which showed that the peri-renal arterial sympathetic nerve fibers have a relatively even distribution along the course of the renal artery, and the median distance and size of the nerve fibers were not significantly different according to the proximity of the segments. The differences in the observed nerve distribution could be multifactorial. Disparities in the study population, methods used in histological preparation, and setting of the measured parameters may have significantly influenced the results. In particular, different tissue harvesting methods may have contributed significantly to the results of the histological analysis. We conducted the current analysis with living human specimens, and ligated and resected proximal renal arteries during the nephrectomy procedure. Consequently, the most proximal part of the renal arteries was excluded from the current analysis, which may have contributed to the distribution pattern of nerve fibers according to the proximity to the aorta.

Although the results may vary depending on the study subject and ablation strategies, previous animal studies indicated that the average lesion depth achieved with currently used catheter-based RDN tools was between 3 to 4 mm^30–32^. Data on the lesion formation by radiofrequency catheter ablation in human subjects are rarely reported due to the inherent limitations of research design. Nevertheless, a human autopsy study involving a patient who underwent RDN reported that effective treatment was delivered only to the superficial nerves within 2 mm in depth, and RF lesions were significantly tapered towards the tissue at a greater depth^33^. A substantial portion of the sympathetic nerve fibers are situated further from the endoluminal surface, and this amount of penetration might be insufficient for complete denervation. Although there is a lack of clinical evidence on the cut off criteria regarding the amount of nerve fiber elimination for successful RDN, our data showed that only 10% of the patients had more than 90% of renal sympathetic nerve fibers located within 2 mm from the renal arteries. This insufficient lesion formation limiting complete and reliable denervation could be a major contributor for the failure of RDN in controlled clinical trials, despite its sound pathophysiological base.

The standard technique of RDN adopted in the SYMPLICITY HTN-3 trial is based on delivering the radiofrequency energy in the main trunk of the renal arteries^34^. However, the utility of this single approach may be limited because of substantial anatomical variations in the renal arteries especially in patients with resistant hypertension^35^. Our data including all consecutive patients who underwent nephrectomy (n = 100) demonstrated that 17% of patients had accessory renal arteries or accessory branched arteries. Aysel *et al*. reported that 27% of patients that underwent donor nephrectomy showed the anatomical variants of renal arteries with accessory arteries and early branching arteries on multi-detector computed tomography^36^. Tarzamni *et al*. showed using multi-detector computed tomography renal angiography that 32.5% of right kidneys vs. 17.1% of left kidneys had an accessory artery and 35.9% of those were early branching^37^. Single‐electrode ablation of the main renal artery has the potential to produce insufficient sympathetic denervation in this population. Preclinical and clinical investigations indicate that extension of the ablation into the distal major renal arterial branches increases the net destruction of sympathetic nerve fibers and improves blood pressure lowering efficacy of RDN^30,38^. However, distal damage to the renal artery might preclude bailout surgical arterial bypass, and safety profiles of distal and branch ablation should be evaluated carefully. The development of more optimal catheter designs and adjustment of treatment algorithms aimed at achieving more complete and reliable renal denervation appears mandatory to enhance the efficacy and safety of RDN.

### Clinical significance of the living human tissue analysis

The key feature of the current analysis is that the peri-arterial anatomy was analyzed using living human tissues. The processes triggered by death and the post-mortem interval may influence the physiological status of the harvested tissue samples, and a previous study proposed significant changes in the number and size of vascular tissues between the living and post-mortem state^22^. In addition, peripheral nerves undergo degenerative changes during the early post-mortem period, which lead to morphological alterations that can be identified in histological evaluations^23^. Consideration should be given to the possibility that these changes may have affected the analysis of the perivascular structures, and it is desirable to verify the post-mortem data using living human samples.

In general, our analysis reaffirmed observations from post-mortem studies that the peri-arterial renal nerve distribution is unsuitable for elimination by the current RDN procedure. We observed a skewed distribution of nerve fibers with a median nerve distance of 1.5 mm, which is relatively shorter than the values from the port-mortem samples^19^. Nevertheless, a significant proportion of nerve fibers were localized beyond the lesion depths created by the current endovascular approach^33^. The fact that our measurements were performed excluding the most proximal segments of the renal arteries, which were reported to have a more distant location of nerves from the arterial lumen, should also be considered. In this regard, the current analysis enriches the body of evidence about the anatomy of human peri-arterial renal nerves and emphasizes the need to develop optimal device-based approaches to modulate the autonomic nervous system.

### Study limitations

There are some inevitable limitations to our study. First, among the patients who underwent radical or simple nephrectomy, there was a relatively small number of intact tissues that could be included in our analysis (n = 28), and our analysis was limited to specimens with single and main renal arteries. A considerable number of patients had accessory renal arteries or accessory branched arteries, and evaluating the innervation pattern in these patients requires further study. Second, comparison of the nerve distribution between the right and left sided kidney was not feasible, because most of the patients underwent unilateral nephrectomy. Third, the majority of the patients underwent radical nephrectomy for malignant indications, and there may be bias in our data of peri-renal arterial nerve distribution due to an accumulation of certain comorbidities. Finally, because this study was evaluated with various CT abdomen protocols, there might be an underestimation of the number of accessory arteries or branches. A previous study reported that CT angiography was superior to CT abdomen or magnetic resonance angiography for the evaluation of the renal vessels. Therefore, in future studies, we will perform the anatomical confirmation of renal artery by analyzing CT angiography of patients who underwent donor nephrectomy.

## Conclusions

We performed histological analysis of the peri-renal arterial sympathetic nervous system with living human samples from elective radical or simple nephrectomy. Within the harvested relative distal renal arterial samples, the overall spatial distribution of the nerve fibers was not significantly different according to the proximity of the renal arterial segments. Another important finding is that a substantial proportion of the sympathetic nerve fibers were located deeper in the peri-arterial soft tissue than in the lesion depth created by the conventional catheter-based RDN system, which is in line with the observations from previous port-mortem studies. Our findings reconfirmed the recently revised understanding of peri-arterial renal autonomic nervous system and enriched the anatomical evidence to overcome the procedural unreliability of the current standard approach for RDN.

## Supplementary information

Supplementary Table 1 to 4, and Supplementary Figure 1 to 4
