# Supplementary material for: Anatomic Conformation of Renal Sympathetic Nerve Fibers in Living Human Tissues
Source: Sci Rep. 2019 Mar 18;9:4831. doi: 10.1038/s41598-019-41159-4 (PMC6423056; doi:10.1038/s41598-019-41159-4)
Supplement: Supplementary file 1 — Supplementary Table 1 to 4, and Supplementary Figure 1 to 4 [file 41598_2019_41159_MOESM1_ESM.docx]

**Anatomic Conformation of Renal Sympathetic Nerve Fibers in Living Human Tissues**

Won-Seok Choe, MD,^1*^ Won Hoon Song^2*^, Chang Wook Jeong^2^, Eue-Keun Choi, MD,^1^ Seil Oh, MD^1^

^1^ Department of Internal Medicine, Seoul National University Hospital, Seoul, Republic of Korea

^2^ Department of Urology, Seoul National University Hospital, Seoul, Korea

^*^equally contributed to this work

**Short title**: Anatomy of the renal sympathetic nervous system

**Corresponding authors**:

Eue-Keun Choi, MD, PhD

Associate professor, Department of Internal Medicine, Seoul National University Hospital,

101 Daehak-ro, Jongno-gu, Seoul, 03080, Republic of Korea

Tel: 82-2-2072-0688

Fax: 82-2-762-9662

E-mail: choiek17@snu.ac.kr

Or

Chang Wook Jeong, MD, PhD

Associate professor, Department of Urology, Seoul National University Hospital,

101 Daehak-ro, Jongno-gu, Seoul, 03080, Republic of Korea

Tel: 82-2-2072-3899

Fax: 82-2-742-4665

E-mail: drboss@korea.com

**Supplementary Materials**

**Contents of the Supplementary Materials**

1. **Supplementary Tables**
2. **Supplementary Figures**

**I. Supplementary Tables**

**Supplementary Table 1. Distribution of peri-renal sympathetic nerves stratified by the size of nerve fibers in proximal, middle, and distal segments of right renal artery (n = 12)**

|  | **Total** | **Segments** | | | |
| --- | --- | --- | --- | --- | --- |
|  | **(n = 1,290)** | **Proximal (n = 389)** | **Middle (n = 417)** | **Distal (n = 484)** | **p value** |
| **Diameter of nerve fiber, μm** |  |  |  |  |  |
| <50 | 524 | 147 | 159 | 218 |  |
| 50-100 | 280 | 82 | 92 | 106 |  |
| 100-150 | 143 | 50 | 52 | 41 |  |
| 150-200 | 85 | 24 | 29 | 32 |  |
| 200-250 | 72 | 20 | 30 | 22 |  |
| 250-300 | 40 | 14 | 10 | 16 |  |
| 300-350 | 36 | 17 | 9 | 10 |  |
| 350-400 | 25 | 8 | 7 | 10 |  |
| 400-450 | 12 | 3 | 3 | 6 |  |
| 450-500 | 6 | 0 | 3 | 3 |  |
| ≥500 | 67 | 20 | 23 | 24 |  |
| **Median diameter, μm** | 66 | 72 | 68 | 58 | 0.858 |

Values are n, % or median. Comparisons of nerve distribution between different segments were performed using a linear mixed model.

**Supplementary Table 2. Distribution of peri-renal sympathetic nerves stratified by the size of nerve fibers in proximal, middle, and distal segments of left renal artery (n = 15)**

|  | **Total** | **Segments** | | | |
| --- | --- | --- | --- | --- | --- |
|  | **(n = 1,639)** | **Proximal (n = 479)** | **Middle (n = 538)** | **Distal (n = 622)** | **p value** |
| **Diameter of nerve fiber, μm** |  |  |  |  |  |
| <50 | 613 | 174 | 202 | 237 |  |
| 50-100 | 528 | 146 | 173 | 209 |  |
| 100-150 | 216 | 66 | 78 | 72 |  |
| 150-200 | 115 | 37 | 38 | 40 |  |
| 200-250 | 66 | 24 | 17 | 25 |  |
| 250-300 | 41 | 14 | 11 | 16 |  |
| 300-350 | 14 | 1 | 8 | 5 |  |
| 350-400 | 17 | 9 | 1 | 7 |  |
| 400-450 | 13 | 3 | 6 | 4 |  |
| 450-500 | 8 | 3 | 3 | 2 |  |
| ≥500 | 8 | 2 | 1 | 5 |  |
| **Median diameter, μm** | 63 | 69 | 63 | 61 | 0.286 |

Values are n, % or median. Comparisons of nerve distribution between different segments were performed using a linear mixed model.

**Supplementary Table 3. Distribution of renal artery by CT abdomen in 100 of patients who underwent nephrectomy (n = 100)**

|  | Mean±SD or Number (%) |
| --- | --- |
| Number of patients | 100 (100%) |
| Right |  |
| Number of main renal artery |  |
| 1 | 100 (100%) |
| Number of accessory renal artery* |  |
| 0 | 90 (90%) |
| 1 | 9 (9%) |
| 2 | 1 (1%) |
| Number of accessory branched artery† |  |
| 0 | 96 (96%) |
| 1 | 4 (4%) |
| Number of total renal artery |  |
| 1 | 87 (87%) |
| 2 | 11 (11%) |
| 3 | 2 (2%) |
| Left |  |
| Number of main renal artery |  |
| 0 | 1†† (1%) |
| 1 | 99 (99%) |
| Number of accessory renal artery* |  |
| 0 | 95 (95%) |
| 1 | 5 (5%) |
| Number of accessory branch artery** |  |
| 0 | 97 (97%) |
| 1 | 3 (3%) |
| Number of total renal artery |  |
| 0 | 1 (1%) |
| 1 | 91 (91%) |
| 2 | 8 (8%) |

* Accessory artery was defined as the sub-artery branched directly from aorta.

† Accessory branched artery was defined as the sub-artery early branched (within 2.0 cm of aorta) from main renal artery.

†† One patient was Lt. nephrectomy status.

**Supplementary Table 4. Measurements of renal artery in study subjects stratified by the history of hypertension**

|  | **Segment** | **Patients without hypertension**  **(n = 12)** | **Patients with hypertension**  **(n = 16)** |  | **p value** |
| --- | --- | --- | --- | --- | --- |
| **Mean lumen diameter,* mm** | Total | 1.81±0.76 | 2.61±1.19 |  | < 0.001 |
|  | Proximal | 1.80±0.86 | 2.50±1.20 |  |  |
|  | Middle | 1.81±0.62 | 2.62±1.25 |  |  |
|  | Distal | 1.82 ±0.71 | 2.72±1.16 |  |  |
| **Lumen area, mm^2^** | Total | 2.76±2.86 | 5.90±5.00 |  | < 0.001 |
|  | Proximal | 2.90±3.49 | 5.70±5.24 |  |  |
|  | Middle | 2.74±2.89 | 5.86±5.15 |  |  |
|  | Distal | 2.62±2.34 | 6.14±4.86 |  |  |
| **Mean EEM diameter, mm** | Total | 2.98±0.79 | 3.98±1.32 |  | < 0.001 |
|  | Proximal | 2.95±0.91 | 3.78±1.30 |  |  |
|  | Middle | 2.96±0.86 | 3.98±1.35 |  |  |
|  | Distal | 3.04±0.66 | 4.18±0.13 |  |  |
| **EEM area, mm^2^** | Total | 7.37±4.65 | 13.44±8.03 |  | < 0.001 |
|  | Proximal | 7.43±5.81 | 12.89±8.27 |  |  |
|  | Middle | 7.27±4.78 | 13.38±8.60 |  |  |
|  | Distal | 7.41±3.52 | 14.04±7.57 |  |  |
| **Mean wall thickness**†**, mm** | Total | 0.57±0.11 | 0.65±0.20 |  | 0.012 |
|  | Proximal | 0.57±0.12 | 0.65±0.20 |  |  |
|  | Middle | 0.56±0.10 | 0.67±0.21 |  |  |
|  | Distal | 0.58±0.11 | 0.65±0.20 |  |  |

Plus-minus values are mean±SD.

* The average of the maximum and minimum luminal diameter.

† The average of the wall thickness from the luminal surface to the EEM measured in 4 quadrants.

Abbreviations: EEM, external elastic membrane

**II. Supplementary Figure**

**Supplementary Figure 1. Distribution of the peri-renal arterial sympathetic nerve fibers in individual patients according to the proximity of the renal arterial segments.**

**
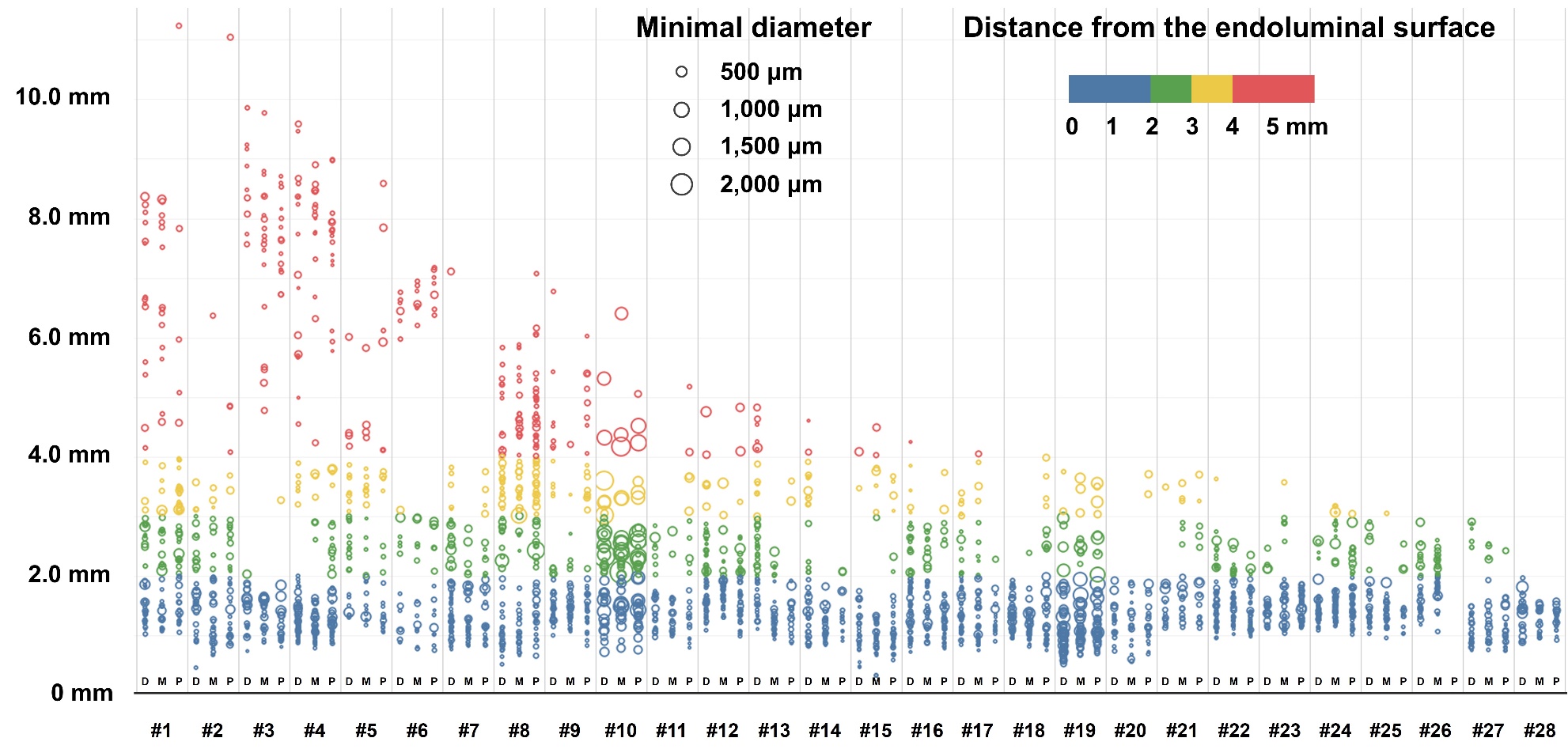
**

The sections separated by vertical lines represent nerve distribution of the distal, middle and proximal segments from the same subject, and the order of the subjects is identical with that in Figure 4.

Abbreviations: D, distal segment; M, middle segment; P, proximal segment.

**Supplementary Figure 2. The overall distribution of sympathetic peri-renal nerve fibers in study subjects stratified by the history of hypertension.**


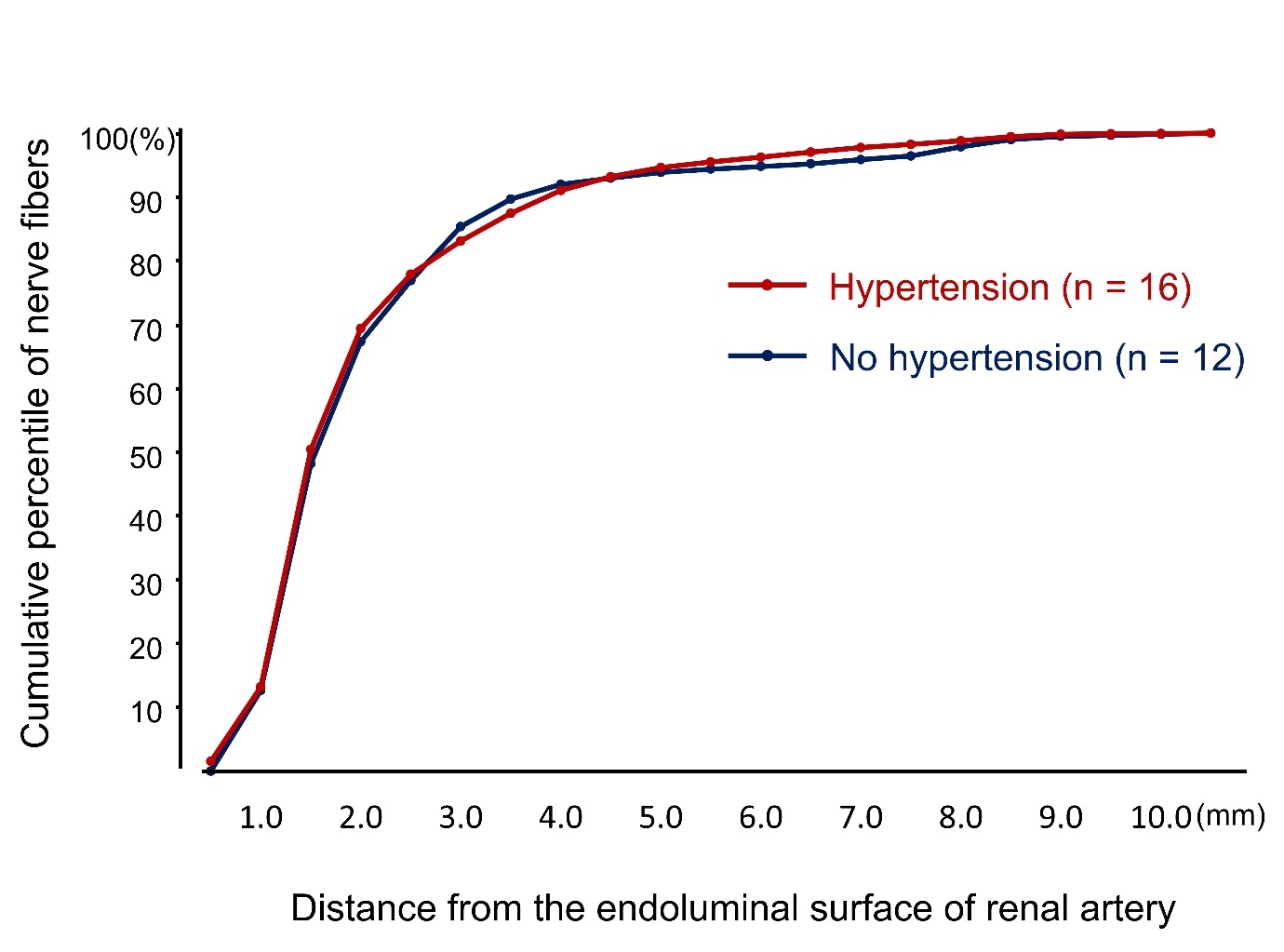


**Supplementary Figure 3. The overall distribution of sympathetic peri-renal nerve fibers in the right and left renal arteries.**


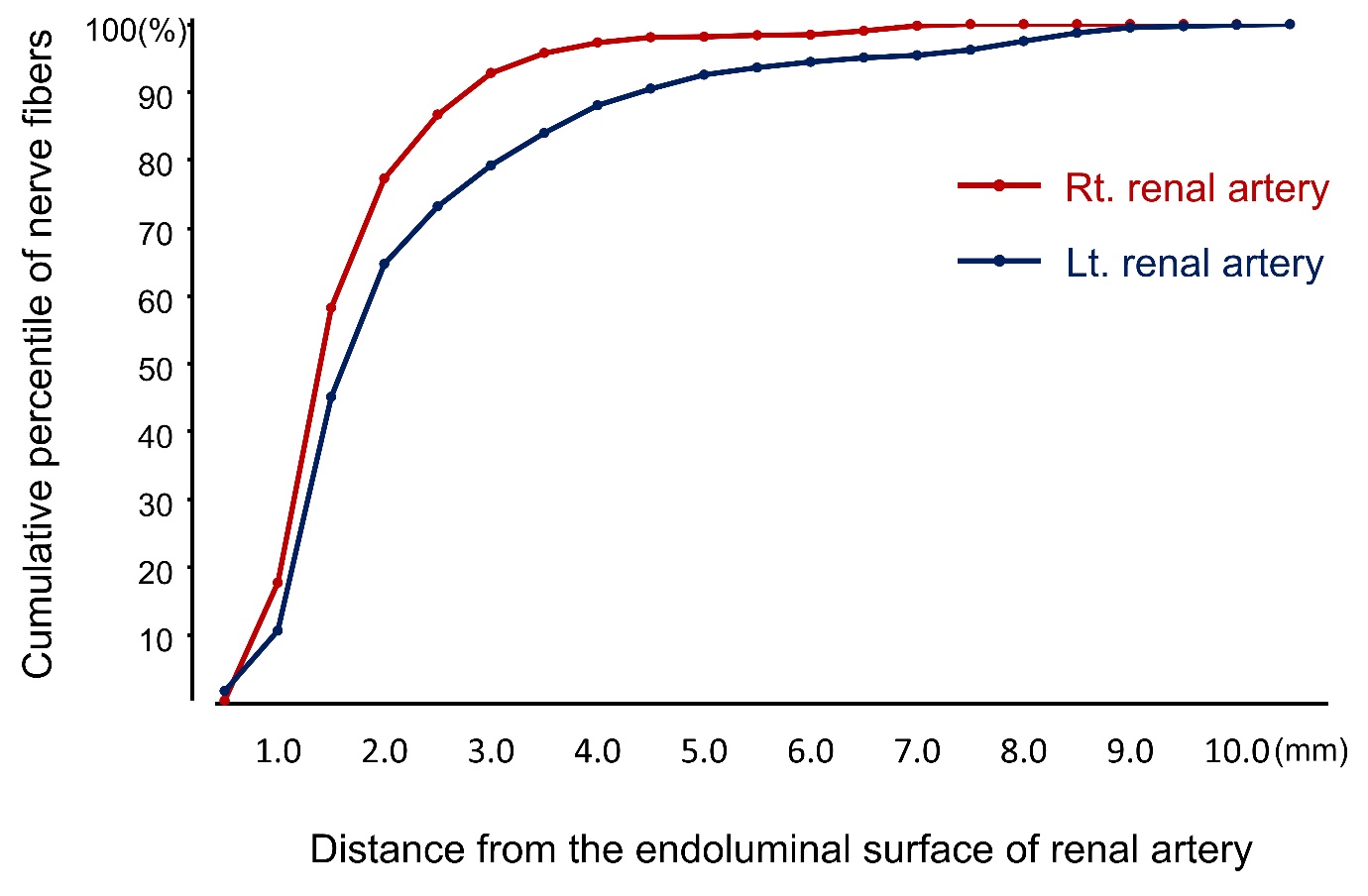


**Supplementary Figure 4. The outer diameter of each segment of main renal artery from the aorta to bifurcation by CT abdomen review 100 of patients who underwent nephrectomy.** (A) The outer diameter of each segment of the main renal artery from bifurcation to 9 mm proximal at 3 mm intervals. (B) The outer diameter of each segment of the main renal artery from the aorta to 21 mm distal at 3 mm intervals.

**A**   **B**
